# Investigation of Tensile Properties of Different Infill Pattern Structures of 3D-Printed PLA Polymers: Analysis and Validation Using Finite Element Analysis in ANSYS

**DOI:** 10.3390/ma15155142

**Published:** 2022-07-25

**Authors:** S. Ganeshkumar, S. Dharani Kumar, U. Magarajan, S. Rajkumar, B. Arulmurugan, Shubham Sharma, Changhe Li, R. A. Ilyas, Mohamed Fathy Badran

**Affiliations:** 1 Department of Mechanical Engineering, Sri Eshwar College of Engineering, Coimbatore 641202, Tamil Nadu, India; ganeshkumar.s@sece.ac.in; 2 Centre for Machining and Material Testing, Department of Mechanical Engineering, KPR Institute of Engineering and Technology, Coimbatore 641407, Tamil Nadu, India; sdharanikumarmech@gmail.com (S.D.K.); enggarul@gmail.com (B.A.); 3 Department of Mechanical Engineering, Bharath Institute of Higher Education and Research, Chennai 600126, Tamil Nadu, India; magarajan84@gmail.com; 4 Department of Mechanical Engineering, Faculty of Manufacturing, Hawassa University, Awassa 3870006, Ethiopia; rajkumar@hu.edu.et; 5 Mechanical Engineering Department, University Center for Research & Development, Chandigarh University, Mohali 140413, Punjab, India; 6 Department of Mechanical Engineering, IK Gujral Punjab Technical University, Main Campus-Kapurthala, Kapurthala 144603, Punjab, India; 7 Department of Mechanical Engineering, Indian Institute of Technology-Ropar, Rupnagar 140001, Punjab, India; 8 School of Mechanical and Automotive Engineering, Qingdao University of Technology, Qingdao 266520, China; sy_lichanghe@163.com; 9 Faculty of Engineering, School of Chemical and Energy Engineering, Universiti Teknologi Malaysia, Johor Bahru 81310, Johor, Malaysia; ahmadilyas@utm.my; 10 Centre for Advanced Composite Materials, Universiti Teknologi Malaysia, Johor Bahru 81310, Johor, Malaysia; 11 Mechanical Engineering, Faculty of Engineering and Technology, Future University in Egypt, New Cairo 11845, Egypt

**Keywords:** poly lactic acid, 3D printing, infill patterns, tensile strength, fusion deposition modeling

## Abstract

The advancement of 3D-printing technology has ushered in a new era in the production of machine components, building materials, prototypes, and so on. In 3D-printing techniques, the infill reduces the amount of material used, thereby reducing the printing time and sustaining the aesthetics of the products. Infill patterns play a significant role in the property of the material. In this research, the mechanical properties of specimens are investigated for gyroid, rhombile, circular, truncated octahedron, and honeycomb infill structures (hexagonal). Additionally, the tensile properties of PLA 3D-printed objects concerning their infill pattern are demonstrated. The specimens were prepared with various infill patterns to determine the tensile properties. The fracture of the specimen was simulated and the maximum yield strengths for different infill structures and infill densities were determined. The results show the hexagonal pattern of infill holds remarkable mechanical properties compared with the other infill structures. Through the variation of infill density, the desired tensile strength of PLA can be obtained based on the applications and the optimal weight of the printed parts.

## 1. Introduction

Rapid prototyping is a time-saving additive manufacturing technique used to reduce wastage and create complex models. It is widely used in the industrial, manufacturing, and development sectors. Three-dimensional(3D) printing can be achieved in both metals and polymer materials, based on the applications. The material infills, support, and layer thickness play a significant role in the strength of the products. Infill structures, such as grids and triangular, hexagonal, triangular, and linear structures are the standard infill structures. Together with the infill structures, infill density also plays a crucial role in the mechanical properties of the 3D-printed objects. The effect of infill density on caries in a PETG part manufactured using the fusion deposition modeling technique was explored. The mechanical properties were found to be significantly influenced by infill structures and density [1,2]. The effect of infill patterns on the mechanical properties was investigated in lightweight 3D-printed PLA cellular parts; the variation of relative flexural modulus concerning the relative density of the material was exhibited in the research. Moreover, this investigation demonstrated the characteristics of infill structures. The hexagonal infill had a higher yield strength compared with square and diagonal structures [3,4,5]. The tensile strength of commercial polymer materials was investigated for the Fused Filament Fabrication 3D-Printing technique. It showed the theoretical and experimental masses of 3D-printed parts for different infill ratios. The specimen with a higher mass possessed good tensile properties [6]. Besides the investigations of mechanical properties with standard infills, topology optimization was carried out to reduce the volume of the material to acquire good load-carrying capacity. The porous structure of the infill was designed based on the local volume of the material. The performance of the bone models and their discrete formulation were compared to honeycomb structures [7,8]. Similar research on mechanical properties was conducted in 3D-printed PLA materials in accordance with the ASTM D638 Type IV Standard, with the yield strength of the specimen tested using a universal testing machine and validated using finite element analysis using ANSYS software [9]. The properties of bioresorbable polymer test specimens with varied infill designs and infill ratios were investigated in biomedical applications. The mechanical properties of commodity implant grade polymers such as PLLA and Lacto flex vary significantly with infill density [10,11]. The mechanical properties of ABS 3D-printed parts were tested concerning the temperature, fill density, velocity of print, and so on. The tensile strength and impact resistance of the specimen were examined and it was demonstrated that the printing time was higher for 100% infills. Hence, the results of the experiment suggest an infill range of less than 100% infill, based on the applications and considering the load parameters [12,13]. Design and topology optimization were carried out for 3D-printed wax patterns for rapid investment casting. A commercial topology optimization tool was utilized in the experimentation to print the wax patterns; patterning of the optimized design was exhibited to reduce the weight of the 3D-printed material [14,15]. Shell–infill composite minimum compliance topology optimization was carried out for additive manufacturing. According to the findings of this study, the numerical technique was linked to the geometry of the object. The composition of non-uniform gradient infill and uniform infill was compared in this research that correlated the relative density and relative stiffness with the infill ratio percentages [16,17]. Investigations of polylactic acid material on its mechanical properties by varying the infill structures we recarried out with triangular, grid, quarter cubic and tri-hexagonal structures. The SEM observations in this study exhibited triangular infill structures produced by raster bonding, which implies good mechanical properties. In triangular infills, a lower number of voids and an absence of racket lines were observed. The results showed that the bonding strength and the optimized infill structures of the layers lead to the good mechanical properties of 3D printed objects [18]. Infill optimization for 3D-printed parts was carried out based on the structural dimensions. This experimentation introduced optimization methodology and, subsequently, integration of global optimization methodology was carried out. A finite element method was applied to the lattice infills and demonstrated that the technique depicted by the results possesses good characteristics in the additive manufacturing process [19,20]. Another approach to shell–infill structures was carried out with the topology optimization technique. The constant-spaced mesh infills were replaced by gradient infills using this technique. The 3D-printed objects possessed remarkable mechanical properties and a reduction in print time was achieved [21,22,23]. Optimal selection of the infill structures was carried out for fusion deposition modeling of PLA materials. The tensile and three-point bending tests reveal the mechanical properties of the PLA-printed parts and showed a significant change in the mechanical properties in terms of print speed, feed rate, and upright orientation [24,25]. Based on previous research on infill structures, the yield strength of the 3D-printed specimen was investigated. Layer thickness and print speed play a significant role in the change in mechanical properties. The common infills are hexagonal, linear, circular, triangular, and linear structures. The development of new structures, such as spherical, rhombic, truncated octahedron, and gyroid structures leads to the creation of a space in the research into the performance of these infills and their mechanical properties. In this research, the mechanical properties of specimens are investigated for gyroid, rhombile, circular, truncated octahedron, and honeycomb structures (hexagon).

Open-work filling has better mechanical properties than solid because it takes less time to print and has better mechanical properties.

The tri-hexagon infill pattern with varied densities of 20%, 40%, and 60% was investigated. A maximum time of 227 min was observed for printing the 60% infill density. The print time increased with infill density. Mechanical properties and printing time were investigated [26]. Ammonium perchlorate-polylactic acid has been successfully 3D printed to test its structural and energetic capabilities. The capabilities of combustible 3D-printing technologies were investigated [27]. Ceramic-filled composite 3D-printed objects were investigated to test their mechanical properties. After the chemical structure modification of zirconium oxide and aluminum oxide ceramic fillers, the material was 3D printed and subjected to mechanical tests [28]. Printing parameters, orientation, raster angle, and materials affected the mechanical properties of fused deposition 3D-printed parts. The proper alterations in the printing parameters led to a significant increase in tensile strength. The factor levels were different from the optimal factor settings [29]. The fused deposition modeling prototypes were investigated using dynamic loading with reinforced composite wires. SiC and Al_2_O_3_ in a Nylon-6 matrix were used as the feedstock filament. The results exhibited an improvement in mechanical properties, such as yield strength, tensile properties, elongation percentage, and Young’s modulus [30]. The 3D-printed specimen was modelled as per ASTM D638 Type IV and subjected to mechanical tests. The ASTM D638 standard is commonly used to test reinforced and non-reinforced plastics. Test samples were placed in the grips of the universal tester at a specified grip separation and pulled until failure [9].

## 2. Materials and Methods

**Figure 1:**
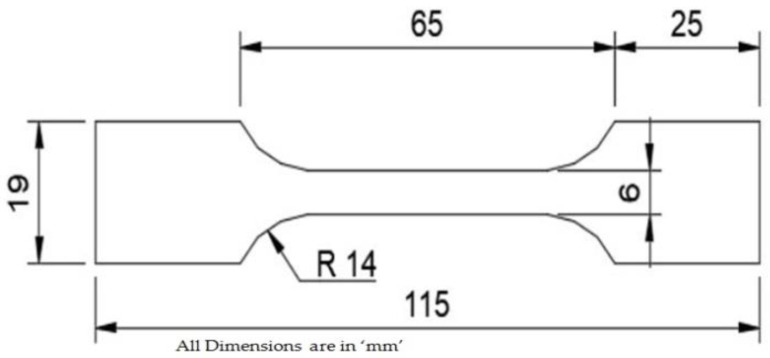
ASTM D638 specimen.

Commercially available Augment 3Di-3D printing (Red) PLA of 1.75 mm diameter was used for the extrusion process. Flash forge Dreamer 3D printer was used to manufacture the test specimens as per ASTM D628 standard to test the tensile properties. The machine was set at a Build Volume: 9.1″× 5.9″× 5.5″ and a layer thickness of 100–500 microns of resolution. A nozzle of 0.4 mm diameter was used to extrude the PLA filament in the manufacturing of the test specimen. A print speed of 200 mm/s and a heated build platform of 120 °C are the maximum ranges for the machine. The maximum positioning precision of the printer was 0.1–0.2 mm. The slicing software Flash Print was used for slicing the part into layers and the creation of infill structures was carried out by this software. For the sliced parts, G-codes were sent to the 3D printer using the software. The dimension of the ASTM D 628 specimen is shown in Figure 1. 

**Figure 2:**
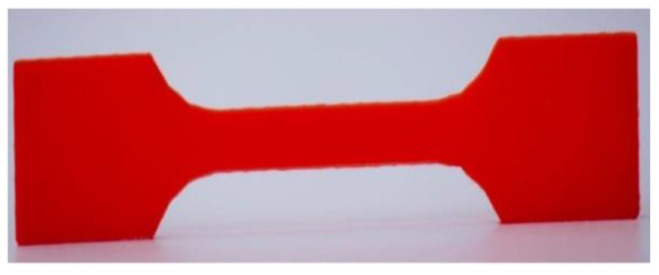
Test Specimen—ASTM D638.

The standard geometry of the tensile test specimen as per ASTM D628 was modeled in Creo Elements. The part file was converted to stereolithographic format (STL) and imported into the Flash Print 3D Printer application for slicing and to send the G-codes to the printer. The static structural module was utilized to create a tensile stress environment. A tensile load of 20.2 MPa was applied on the two sides of the specimen [31,32]. The local stresses were simulated to investigate the mechanical properties of the specimen with gyroid, rhombile, circular, truncated octahedron, and honeycomb structures (hexagonal) patterns with varied infill ratios. The infill structures were designed in Fusion 360 with various infill densities and exported to flash print and ANSYS applications for manufacturing the specimen and evaluating its mechanical properties, respectively. In our additive manufacturing process, 120 °C was maintained and an extrusion temperature of 210 °C was maintained in the extruder to melt the PLA filament. A print speed of 60 mm/s and a layer thickness of 0.18 mm were maintained throughout the manufacturing of the specimen. This layer thickness was consistently maintained for all varied percentages of infill density and infill structures, with the first layer of thickness of the specimen being maintained at 0.27 mm. The structure’s infill was developed in Fusion 360. It was tested on a universal testing machine. The specimen was loaded into the testing machine jaws and hydraulic loads were applied between the two ends of the specimen. The loads were applied gradually until the specimen fractured due to the applied tensile loads. The 3D-printed PLA specimen is shown in Figure 2.

### 2.1. Printer Setting

**Figure 3:**
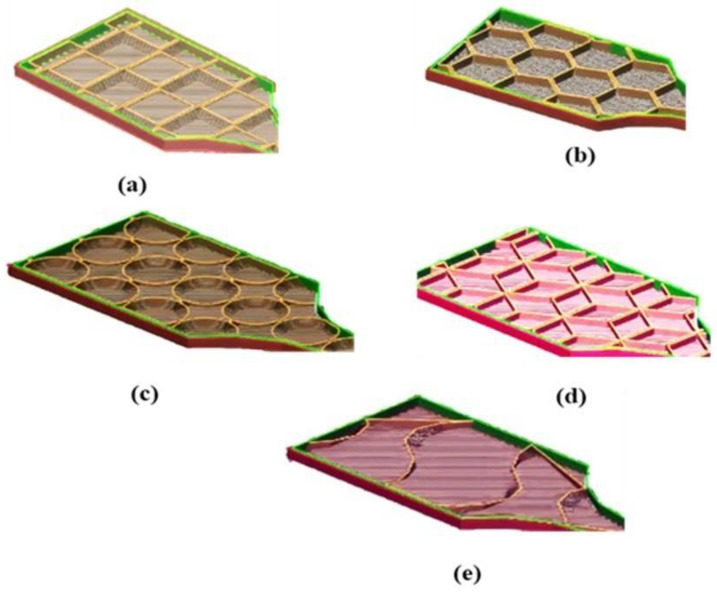
(**a**) Truncated octahedron infill structure, (**b**) Hexagonal infill structure. (**c**) Circular infill structure, (**d**) Rhombile infill structure, (**e**) Gyroid infill structure.

The material density was taken as 1.24 kg/cm^3^ for polylactic acid. In printer settings, the specimen is in the boundary regions of the platform with 0° of orientation for printing the specimen without supports. The infill structure is shown in Figure 3. For finite element analysis, a specimen 3D model was imported in IGEs format and yield stress and fracture growth were analyzed using static structural and explicit dynamics analysis. In the preprocessor, polylactic acid material was assigned a Young’s modulus of 13.8 GPa and a density of 1.24 kg/cm^2^ was given to calculate the mechanical properties [33]. Automatic mesh generation was selected with a mesh relevance of 2. In explicit dynamics analysis settings, one end of the specimen was fixed and the other end of the specimen was placed on the displacement constraint that replicates the universal testing machine jaws. An end time of 0.001 s was set and a displacement of 10 mm was assigned. It was assumed that the fracture growth and failure of the specimen would occur within a distance of 10 mm of displacement. The stress concentration, strain, and energy absorbed by the specimen were analyzed in the ANSYS workbench for hexagonal (honeycomb), rhombile, truncated octahedron, gyroid and spherical infill structures [33,34].

### 2.2. Tensile Test 

**Figure 4:**
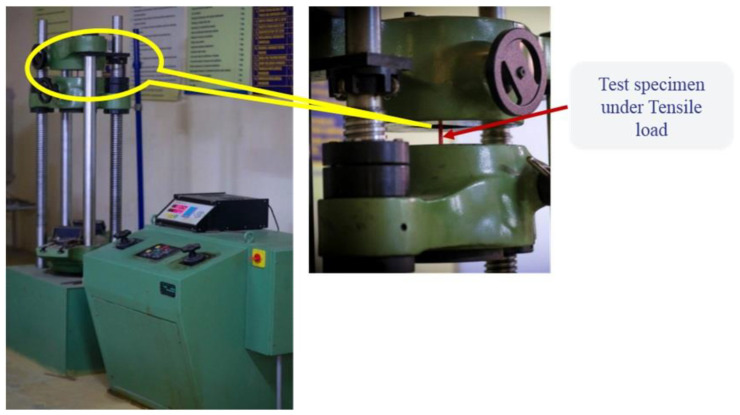
Universal testing machine with specimen.

The specimen was loaded in the universal testing machine and the yield strength was observed for the five different infill structures, namely gyroid, rhombic, spherical and honeycomb structures (hexagonal). The displacement of the crosshead was achieved by the hydraulic system, and the pressure applied was noted at the fracture point of the specimen. The tensile strength of the specimen was taken from the pressure at the time of failure of the specimen in the testing crossheads. The ASTM D 638 specimen was loaded with various infill structures with varying infill rations, and the pressure at the specimen failure was recorded [35,36]. The fixed crosshead and movable crossheads of the universal testing machine and the loading of the specimen on the machine are exhibited in Figure 4.

## 3. Results and Discussion

**Figure 5:**
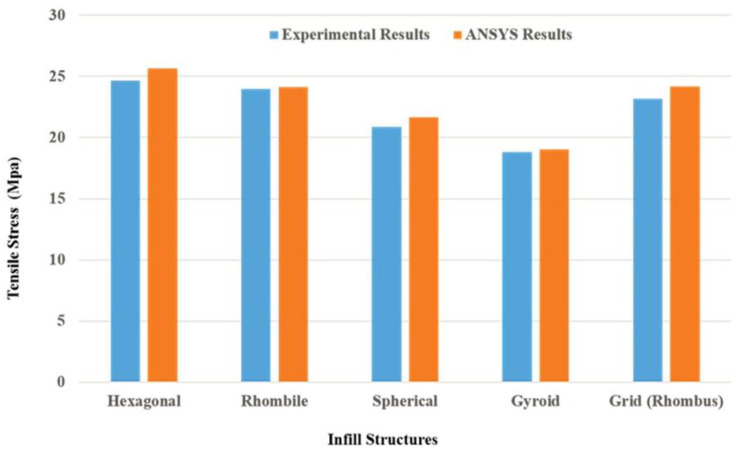
Tensile stress for various infill structures.

The tensile properties were observed in the universal testing machine through fracture development and failure of the material. It was observed that the specimen can withstand a 31.69 MPa tensile load. The maximum yield strength of the specimen was greater irrespective of the infill structure. Similarly, increasing the infill ratio of the specimen reduced the deviation of yield strength [37,38,39]. This phenomenon demonstrates thatthe increment in infill ratio leads to an increase in the yield strength, which is replicated in the ANSYS results. The variation of tensile stress for various infill structures are exhibited in Figure 5. 

This indicates the similarity of yield stress observed in the hexagonal infill structure (honeycomb) which possesses 13.79 MPa of ultimate tensile strength. From other infills, it was found that the hexagonal infill structure holds good tensile properties. The pattern of increasing yield strength is observed in all infill structures proportionately to the weight of the material being increased. Spherical and gyroid infill structures show lower tensile values due to the infill material bonding strength [40,41]. The area of contact between the patterns in the infill is smaller compared with that of the honeycomb structures. This phenomenon leads to a decrease in bond strength and the lowest tensile strength. The gyroid structure infill has 8.56 MPa of ultimate tensile strength for the same infill ratio, while the hexagonal structures possess 13.79 MPa of yield strength [42].

**Figure 6:**
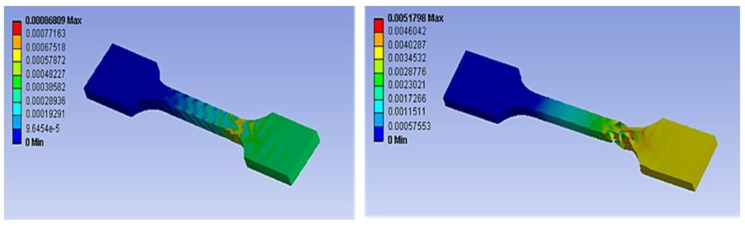
Finite element results.

The fracture development of the ASTM D 638 specimen was simulated in ANSYS. The development stages are shown in the figure. The mechanical stresses acting on the specimen at one end lead to an increase in stress at the weaker regions [43]. The tensile load is transformed from the layers to the inner layers of the infill. The bonding of the infill is perpendicular to the direction of the tensile force applied since the orientation of the specimen in printing is 0°. Finite element results of a hexagonal infill specimen are shown in Figure 6.

**Figure 7:**
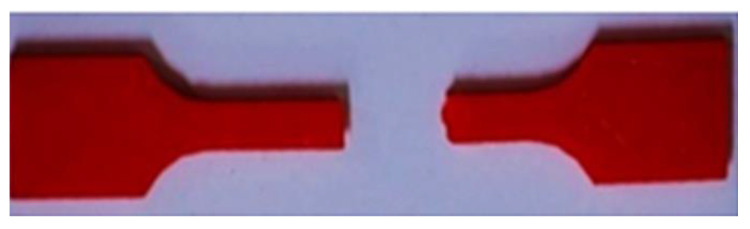
3D printed ASTMD638 specimen after failure.

The fracture of the specimen is due to the breaking of bonds in the infill structures. From the observations, the hexagonal infill structure carries good tensile properties due to its high bonding area [44,45]. The 3D-printed ASTMD638 specimen after failure is shown in Figure 7. The results show that the fracture growth is from the infill bonds and, after increasing the pressure, failure occurs in the bonding areas. Fracture is observed at the neck region of the specimen.

**Figure 8:**
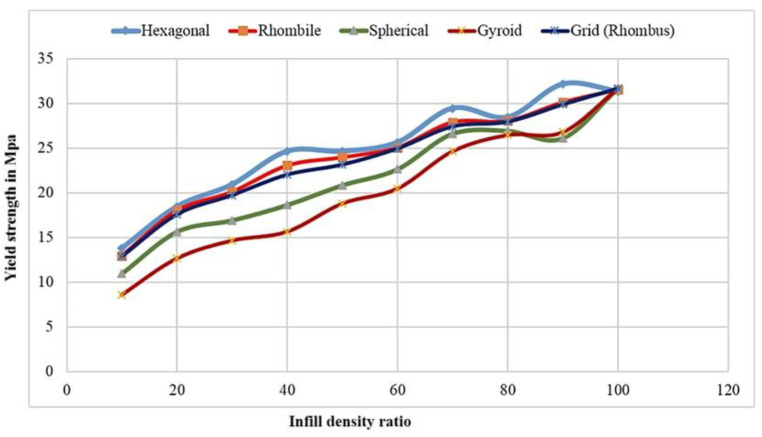
Maximum yield strength obtained in universal testing machine and infill ratio for varied infill structures.

**Figure 9:**
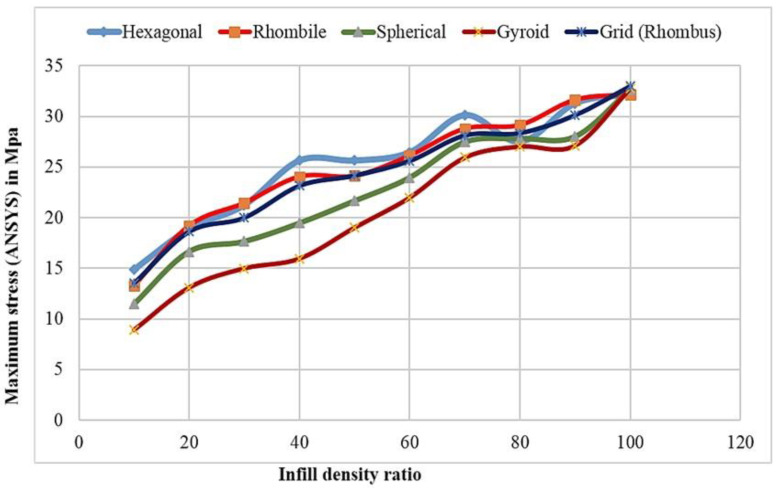
Maximum yield strength obtained in ANSYS workbench and infill ratio for varied infill structures.

The relation between the maximum yield strength obtained from the tensile tests and the ANSYS results are shown in Figure 8 and Figure 9, respectively. It was observed that irrespective of the infill structures (gyroid, rhombic, spherical, and honeycomb), the yield strengths are closer to each other since the material ratio increases in the infill structures: the yield strength is proportional to the bonding layers and leads to an increase in the mechanical properties of the material.

**Figure 10:**
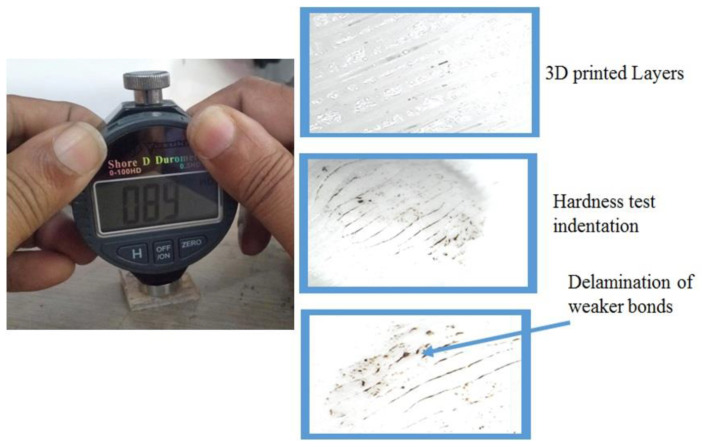
Hardness test setup and delamination of weaker bonds of 3D-printed test specimen.

The hardness of the 3D-printed specimen was measured using a Shore D hardness testing machine with a measuring range of 0.5–100 HD. The hardness test setup and microscopic image of the indentation are exhibited in Figure 10. A test load of 0–45 N was applied to the test specimen, which was fixed to a fixed slab. The indenter needle was pressed during the test for 1 s. Hardness values of 46 HD, 56 HD, 61 HD, and 84 HD were observed in PLA 3D-printed specimens with 20%, 40%, 60%, and 80% infills, respectively. The 80% infill ratio showed a superior shore D hardness value compared with other specimens. However, the increased hardness value in this sample was mainly due to the large volume of material filled in layer by layer.

## 4. Conclusions

In this experimentation, the yield strength of the infill structures and density ratio were investigated. The test results were validated using finite element analysis and, based on the values, yield stress, local stresses, and fracture development concerning the infill density and infill structures were tabulated. From the results, optimal weight, infill ratio, and infill structures could be selected based on the requirements of tensile strength applications. Infill patterns for gyroid, rhombic, spherical, and honeycomb structures (hexagon) were3D modelled and sliced using Flash print and Autodesk Fusion 360 slicing software. The specimen was manufactured as per ASTMD638 and tested on a universal testing machine to examine its tensile properties. The findings are supported by previous research and ANSYs finite element analysis. The relationship between the infill ratio and infill structures with the yield strength is demonstrated in this investigation. The hexagonal (honeycomb) infill structure possesses good tensile behavior compared with other infill rations. Moreover, it is observed that the printing time increases with respect to the infill ratio. The printing time of the specimen is reduced at a 100% infill ratio since the reduction in travel time of the extruder compensates for the reduction in manufacturing time.

## Data Availability

No data were used to support this study.

## References

[1] Srinivasan R., Ruban W., Deepanraj A., Bhuvanesh R., Bhuvanesh T. (2020). Effect on infill density on mechanical properties of PETG part fabricated by fused deposition modelling. Mater. Today Proc..

[2] Caivano R., Tridello A., Paolino D., Chiandussi G. (2020). Topology and fibre orientation simultaneous optimisation: A design methodology for fibre-reinforced composite components. Proc. Inst. Mech. Eng. Part L J. Mater. Des. Appl..

[3] Lubombo C., Huneault M.A. (2018). Effect of infill patterns on the mechanical performance of lightweight 3D-printed cellular PLA parts. Mater. Today Commun..

[4] Hlinka M. (2020). Non-destructive Testing for the Influence of Infill Pattern Geometry on Mechanical Stiffness of 3D Printing Materials. Ph.D. Thesis.

[5] Ranjan R., Ayas C., Langelaar M., van Keulen F. (2020). Topology Optimisation Techniques. Precision Metal Additive Manufacturing.

[6] Tanikella N.G., Wittbrodt B., Pearce J. (2017). Tensile strength of commercial polymer materials for fused filament fabrication 3D printing. Addit. Manuf..

[7] Wu J., Clausen A., Sigmund O. (2017). Minimum compliance topology optimization of shell–infill composites for additive manufacturing. Comput. Methods Appl. Mech. Eng..

[8] Gupta P., Krishnamoorthy B., Dreifus G. (2020). Continuous toolpath planning in a graphical framework for sparse infill additive manufacturing. Comput. Aided Des..

[9] Kumar S.A., Narayan Y.S. (2019). Tensile testing and evaluation of 3D-printed PLA specimens as per ASTM D638 type IV standard. Innovative Design, Analysis and Development Practices in Aerospace and Automotive Engineering (I-DAD 2018).

[10] Culbreath C.J., Gaerke B., Taylor M.S., McCullen S.D., Mefford O.T. (2020). Effect of Infill on Resulting Mechanical Properties of Additive Manufactured Bioresorbable Polymers for Medical Devices. Materialia.

[11] Alhazmi M.W., Backar A.H. (2020). Influence of Infill density and Orientation on the Mechanical Response of PLA+ Specimens Produced using FDM 3D Printing. Int. J. Adv. Sci. Technol..

[12] Alvarez C., Kenny L., Lagos C., Rodrigo F., Aizpun M. (2016). Investigating the influence of infill percentage on the mechanical properties of fused deposition modelled ABS parts. Ing. E Investig..

[13] Li Z., Wang L., Ma G. (2020). Mechanical improvement of continuous steel microcable reinforced geopolymer composites for 3D printing subjected to different loading conditions. Compos. Part B Eng..

[14] Wang J., Sama S.R., Lynch P.C., Manogharan G. (2019). Design and topology optimization of 3D-Printed wax patterns for rapid investment casting. Procedia Manuf..

[15] Yang C., Xu P., Xie S., Yao S. (2019). Mechanical performances of four lattice materials guided by topology optimisation. Scr. Mater..

[16] Wu J., Aage N., Westermann R., Sigmund O. (2017). Infill Optimization for Additive Manufacturing—Approaching Bone-Like Porous Structures. IEEE Trans. Vis. Comput. Graph..

[17] Kain S., Ecker J.V., Haider A., Musso M., Petutschnigg A. (2020). Effects of the infill pattern on mechanical properties of fused layer modeling (FLM) 3D printed wood/polylactic acid (PLA) composites. Eur. J. Wood Wood Prod..

[18] Aloyaydi B., Sivasankaran S., Mustafa A. (2020). Investigation of infill-patterns on mechanical response of 3D printed poly-lactic-acid. Polym. Test..

[19] García-Domínguez A., Claver J., Sebastián M.A. (2019). Infill optimization for pieces obtained by 3D printing. Procedia Manuf..

[20] Schmitt M., Mehta R.M., Kim I.Y. (2020). Additive manufacturing infill optimization for automotive 3D-printed ABS components. Rapid Prototyp. J..

[21] Qiu W., Jin P., Jin S., Wang C., Xia L., Zhu J., Shi T. (2020). An evolutionary design approach to shell-infill structures. Addit. Manuf..

[22] Alexandersen J., Andreasen C.S. (2020). A review of topology optimisation for fluid-based problems. Fluids.

[23] Al-Tamimi A.A., Almeida H., Bartolo P. (2020). Structural optimisation for medical implants through additive manufacturing. Prog. Addit. Manuf..

[24] Chacón J.M., Caminero M.A., García-Plaza E., Núnez P.J. (2017). Additive manufacturing of PLA structures using fused deposition modelling: Effect of process parameters on mechanical properties and their optimal selection. Mater. Des..

[25] Carneau P., Mesnil R., Roussel N., Baverel O. (2020). Additive manufacturing of cantilever-From masonry to concrete 3D printing. Autom. Constr..

[26] Maurya S., Malik B., Sharma P., Singh A., Chalisgaonkar R. (2022). Investigation of different parameters of cube printed using PLA by FDM 3D printer. Mater. Today Proc..

[27] Monogarov K.A., Fomenkov I.V., Pivkina A.N. (2022). FDM 3D printing of combustible structures: First results. Mendeleev Commun..

[28] Nakonieczny D.S., Kern F., Dufner L., Antonowicz M., Matus K. (2021). Alumina and Zirconia-Reinforced Polyamide PA-12 Composites for Biomedical Additive Manufacturing. Materials.

[29] Doshi M., Mahale A., Singh S.K., Deshmukh S. (2021). Printing parameters and materials affecting mechanical properties of FDM-3D printed Parts: Perspective and prospects. Mater. Today Proc..

[30] Singh R., Singh N. (2017). Effect of hybrid reinforcement of SiC and Al2O3 in Nylon-6 matrix on mechanical properties of feed stock filament for FDM. Adv. Mater. Process. Technol..

[31] Saniman M.N.F., Bidin M.F., Nasir R.M., Shariff J.M. (2020). Flexural Properties Evaluation of Additively Manufactured Components with Various Infill Patterns. Int. J. Adv. Sci. Technol..

[32] Tho N.H., Minh T.C., Tai N.P. (2020). The effect of infill pattern, infill density, printing speed and temperature on the additive manufacturing process based on the FDM technology for the hook-shaped components. J. Polimesin.

[33] Kuppuswamy H., Ganesan A. (2016). Structural, mechanical and in vitro studies on pulsed laser deposition of hydroxyapatite on additive manufactured polyamide substrate. Int. J. Bioprinting.

[34] Zai B.A., Khan M.A., Khan S.Z., Asif M., Khan K.A., Saquib A.N., Mansoor M.S., Mujtaba A. (2020). Prediction of crack depth and fatigue life of an Acrylonitrile Butadiene Styrene cantilever beam using dynamic response. J. Test. Eval..

[35] Kumar M.V., Padmanaban G., Balasubramanian V. (2020). Role of tool pin profiles on wear characteristics of friction stir processed magnesium alloy ZK60/silicon carbide surface composites. Mater. Und Werkst..

[36] Eutionnat-Diffo P.A., Chen Y., Guan J., Cayla A., Campagne C., Zeng X., Nierstrasz V. (2019). Stress, strain and deformation of poly-lactic acid filament deposited onto polyethylene terephthalate woven fabric through 3D printing process. Sci. Rep..

[37] Song Y., Li Y., Song W., Yee K., Lee K.Y., Tagarielli V.L. (2017). Measurements of the mechanical response of unidirectional 3D-printed PLA. Mater. Des..

[38] Al-Tamimi A.A., Quental C., Folgado J., Peach C., Bartolo P. (2020). Stress analysis in a bone fracture fixed with topology-optimised plates. Biomech. Modeling Mechanobiol..

[39] Saniman M.N.F., Hashim M.H.M., Mohammad K.A., Wahid K.A.A., Muhamad W.M.W., Mohamed N.H.N. (2020). Tensile Characteristics of Low Density Infill Patterns for Mass Reduction of 3D Printed Polylactic Parts. Int. J. Automot. Mech. Eng..

[40] Reddy A.P., Krishna P.V., Rao R.N. (2018). Mechanical and Wear Properties of Aluminum-Based Nanocomposites Fabricated through Ultrasonic Assisted Stir Casting. J. Test. Evaluation.

[41] Ball D.L., Martinez M., Baldassarre A., Dubowski D.M., Carlson S.S. (2020). Analytical and Experimental Investigation of Elastic–Plastic Strain Distributions at 2-D Notches. J. Test. Eval..

[42] Vigneshkumar M., Padmanaban G., Balasubramanian V. (2018). Influence of Tool Rotational Speed on the Formation of Friction Stir Processing Zone in Cast Zk60/SiCp Magnesium Alloy Surface Composites. Mater. Perform. Charact..

[43] Nemati R., Dave E.V., Sias J.E. (2020). Statistical evaluation of the effects of mix design properties on performance indices of asphalt mixtures. J. Test. Eval..

[44] Parab S., Zaveri N. (2020). Investigating the Influence of Infill Pattern on the Compressive Strength of Fused Deposition Modelled PLA Parts. Proceedings of the International Conference on Intelligent Manufacturing and Automation.

[45] Thirumalaikumarasamy D., Balasubramanian V., Sabari S.S. (2017). Prediction and optimization of process variables to maximize the Young’s modulus of plasma sprayed alumina coatings on AZ31B magnesium alloy. J. Magnes. Alloy..

